# Translation stalling in neurons: a critical mechanism for timely protein delivery to distal cellular processes

**DOI:** 10.1042/BST20253066

**Published:** 2025-08-26

**Authors:** Jingyu Sun, Lily Drever, Joaquin Ortega, Wayne S. Sossin

**Affiliations:** 1 Department of Anatomy and Cell Biology, McGill University, Montreal, Quebec, H3A 0C7, Canada; 2 Centre de Recherche en Biologie Structurale, McGill University, Montreal, Quebec, H3G 0B1, Canada; 3 Department of Neurology and Neurosurgery, Montreal Neurological Institute, McGill University, Montreal, Quebec, H3A 2B4, Canada

**Keywords:** Cryo-EM, FRMP, neuronal RNA granule, ribosome, stalled polysomes, translational control

## Abstract

Neurons require local protein synthesis at synapses to control their proteome in response to local inputs. Work over the past two decades has revealed that neurons can use a specialized mechanism to transfer mRNAs and ribosomes to local sites in addition to canonical mechanisms used in many cell types. Neurons initiate translation on the ribosomes in the cellular soma, pause the process, and then package these stalled ribosomes into structures known as ‘neuronal RNA granules’ that are transported to synapses. This review provides an overview of recent studies that characterize these ribosomes/granules biochemically and structurally. These studies provide novel insights into the unique and specialized characteristics of neuronal ribosomes that facilitate this distinct transport mechanism. Many questions remain, including the influence of mRNA sequences on the stalling process and how ribosomes in the granules avoid the physiological responses that, in other cells, recycle ribosomal subunits upon stalling. Many neurodevelopmental disorders, such as autism and intellectual disability, occur when local translation is disrupted in neurons. Understanding mechanisms underlying the stalling of neuronal ribosomes, their transport to processes, and their reactivation may enable novel therapies for neurodevelopmental diseases.

## RNA transport and local translation in cells

Most cells translate proteins close to where they will be used by delivering mRNAs from the nucleus to specific locations in the cytoplasm where the protein is required [1,2]. To accomplish RNA transport, RNA-binding proteins (RBPs) bind to either the coding sequence or the 5′ or 3′ untranslated regions of the mRNA, forming mRNA-containing ribonucleoproteins (mRNPs) [3]. mRNPs then interact indirectly through adaptor proteins, or directly with motor proteins, microtubules, and actin filaments, to be transported to specific locations within the cell where they are translated [4]. mRNP transport can also occur through tethering or hitchhiking on membrane-enclosed organelles such as endosomes or mitochondria [5–7].

Local translation offers benefits to the cell. It can generate sites of high protein concentration at specific sites without requiring the protein to be expressed and transported throughout the entire cell. Synthesizing proteins close to their site of action circumvents undesired interactions with the newly synthesized protein during transit. Since one mRNA can act as a template for up to 1000 protein copies, it may conserve energy to transport a few mRNA molecules instead of a large number of proteins [2]. Finally, local translation confers the ability to control protein production locally near the site where the proteins are needed [2,8].

**Figure 1:**
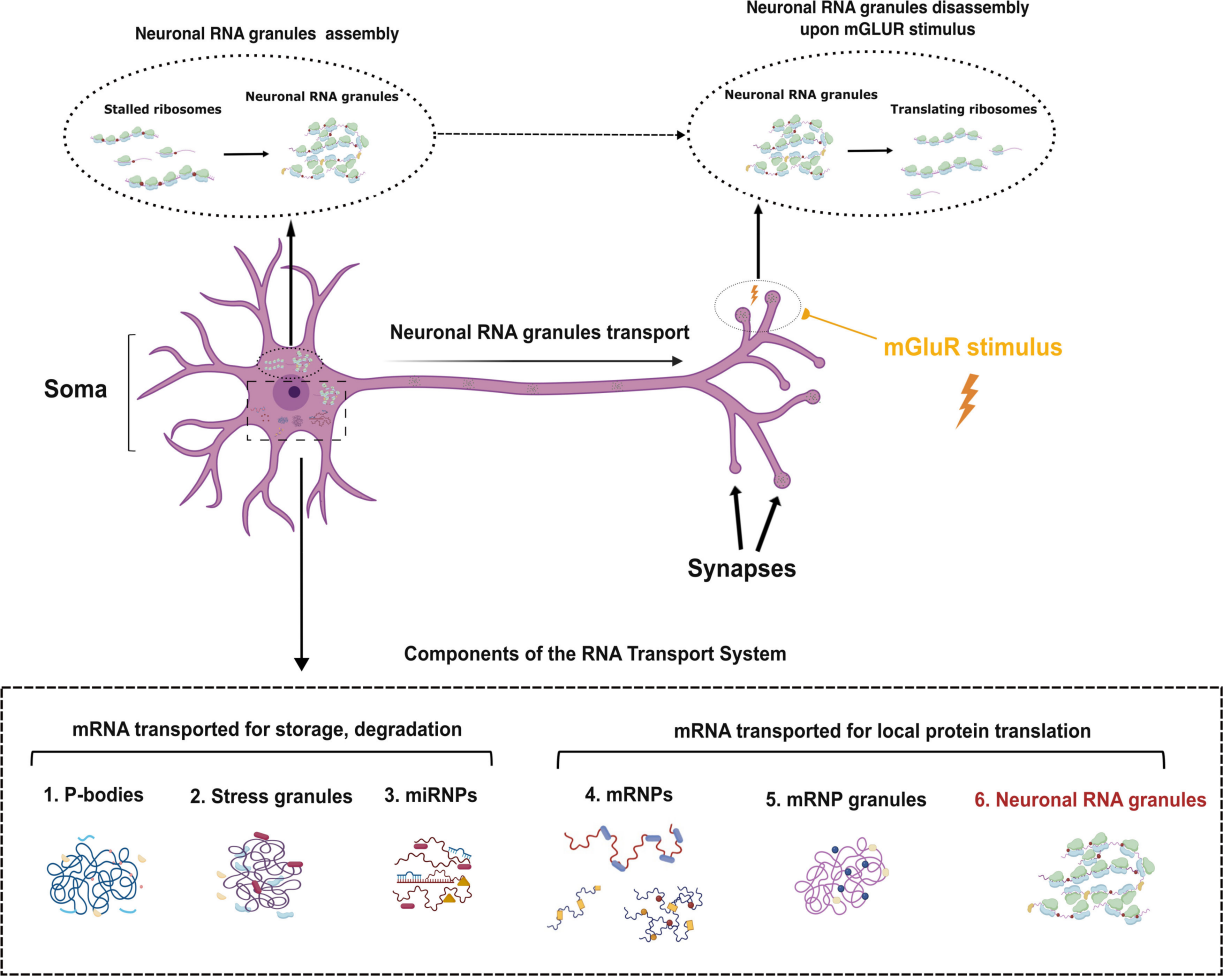
Diagram of a neuron architecture and the RNA transport system. The diagram shows the architecture of a neuron, highlighting soma, axon, and synapses, where neuronal RNA granules assemble, transport, and disassemble, respectively. The granules are also transported in dendrites that are not highlighted in the figure. The dashed circles indicate that neuronal RNA granules are formed by stalled ribosomes at the soma during assembly and reactivated for disassembly at synapses in response to mGluR stimulus. The dashed box outlines the major components of RNA transport system that are present in all cells, and the components that are likely to be transported in neuronal processes to synapses. mGluR, metabotropic glutamate receptor; P bodies, processing bodies.

While mRNA localization and local translation are universal characteristics of cells, neurons face particular challenges, given their highly polarized morphology. Many dendrites radiate outward from the neuronal cell body, forming a dense arbor that expands tens of millimeters. Neurons also present a single axon that can extend from a few to hundreds of centimeters in length (Figure 1). At long distances from the soma, dendrites and axons form active synapses with thousands of other neurons to form neuronal circuitry. Generating and retaining functional neuronal circuits requires regulation of local proteomes and proteostasis through local protein synthesis that is independent of the cell soma. Breakdowns in this ability are a common theme in neurodegenerative diseases [9–11].

Neurons, similar to other cells, assemble their mRNA into mRNPs that are deployed to neuronal processes through cytoskeletal motors [12]. Neurons also assemble large structures known as ’neuronal RNA granules’ for transporting mRNAs to processes[13,14]. There are many RNA granules that play roles in RNA transport and processing (Figure 1). These granules undergo phase separation due to the presence of many mRNAs and RBPs with disordered domains that facilitate liquid–liquid phase separation [15,16]. Processing bodies (P bodies) are sites for the storage and degradation of repressed mRNAs ; [17,18]. mRNAs repressed by miRNAs can form their own particles called miRNPs (Figure 1) or be stored in P bodies [19,20]. Stress granules contain mRNA stalled during initiation, often due to phosphorylation of elF2α [21]. Neurons form miRNPs, P bodies, and stress granules, but it is not clear if they are used to transport mRNAs to processes. While neuronal RNA granules share some RBPs with miRNAs, P bodies, and stress granules, they are unique in containing 80S ribosomes [13,14,22–24] (Figure 1). Some transported mRNPs in neurons contain a single mRNA blocked at translation initiation and are not associated with 80S ribosomes. It remains unclear whether the mRNPs blocked at initiation accumulate into large mRNP-specific granules (Figure 1) or if they are also found in neuronal RNA granules along with 80S ribosomes, or both. It is also not known which transport mechanisms (such as direct attachments to microtubule motors or hitchhiking on transported organelles) are used to transport either mRNP-specific granules or 80S-containing granules within processes.

Although the existence of neuronal RNA granules containing 80S ribosomes has been recognized since the 1990s [25], recent studies have helped us understand that these RNA granules contain specialized ribosomes engaged in protein synthesis [26–28] but stalled in a manner that inhibits ribosome recycling. These stalled ribosomes are reactivated after transport for protein synthesis to support synaptic function [29].

Despite recent research, we still do not fully understand the fundamental structural and mechanistic aspects of these stalled ribosomes. What distinctive composition, structure, and functional features in these ribosomes lead to their susceptibility to translation stalling? It is possible that ribosomes incorporated into neuronal RNA granules are already synthesized with unique characteristics during normal biogenesis in the cellular soma. However, it is also possible that they are further modified upon delivery to the end of the neuronal extensions. Recent studies provide evidence that axonally synthesized ribosomal protein isoforms can join pre-existing ribosomes in these axons, contributing to additional ribosome specialization [30,31]. The contribution of the mRNA sequences read by these ribosomes to the process of stalling is also not known. Furthermore, how do these ‘specialized’ stalled ribosomes differ from ‘regular’ ribosomes that may have stalled during protein synthesis for other reasons, such as tandem rare codons or oxidized mRNA, and are subsequently targeted for recycling?

This review summarizes recent findings that help address some aspects of these important questions. However, we remain far from achieving a complete understanding of the relevance and role of these RNA granules in both normal and pathological neuronal physiology [32–34].

## Neuronal RNA granules are clusters of condensed translationally arrested ribosomes

**Figure 2:**
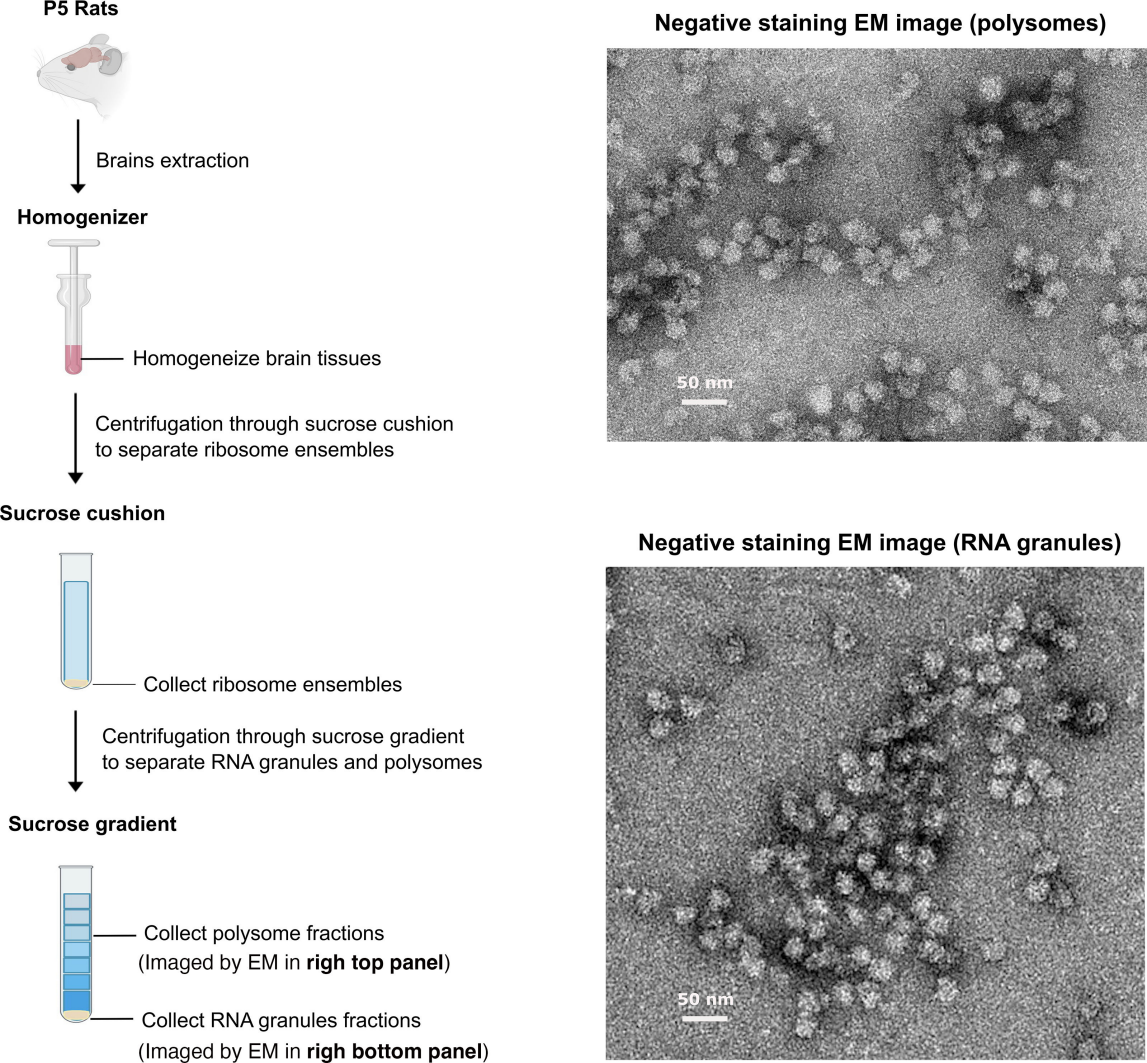
Visualization of RNA granules and polysomes with negative staining electron microscopy. The RNA granules and polysomes are purified from 5-day old rat brains using sucrose gradient ultracentrifugation (left panel). The electron micrographs (right panel) show regular polysomes (top) and neuronal RNA granules (bottom) purified from neurons after applying negative staining. Samples in these images were prepared and imaged as described in our previous publication [27].

Neuronal RNA granules were first visualized in neuronal processes using SYTO 14, a cell-permeant fluorescent dye whose emission intensity is enhanced upon RNA binding, and correlative light-electron microscopy experiments demonstrating that the fluorescent granules corresponded to clusters of ribosomes that also contained poly(A^+^) mRNA [25]. Subsequent studies were able to purify these clusters of ribosomes from neuronal RNA granules using sucrose gradient ultracentrifugation (a standard technique to purify ribosomes) [13,35,36]. In these gradients, clusters of ribosomes from RNA granules sediment because they are denser and heavier, making them easy to separate from monosomes and polysomes [36,37] (Figure 2). Their overall sedimentation behavior also differs significantly from that of polysomes. Treatments that do not affect polysome sedimentation, such as high salt and ionic detergents, disrupt the sedimentation of ribosome clusters found in neuronal RNA granules, indicating they have different overall organization and properties from polysomes [36,37]. Ribosome clusters similar to those in neuronal RNA granules have not been observed outside the nervous system.

## RBPs are an integral part of neuronal RNA granules

Proteomics was used to analyze the protein composition of the ribosome clusters enriched by sucrose gradient ultracentrifugation methods (Figure 2). Three separate studies [35,36,38] demonstrated that these ribosome clusters contain abundant levels of specific RBPs, including G3BPs, Staufens, PABPs, Purs, UPF1, FMRs, ELAVs, Caprin, nucleolin, and hnRNP proteins, particularly hnRNPQ (Syncrip) and hnRNPU. Moreover, in neuronal processes, many of these RBPs are found in large puncta containing 80S ribosomes [26,35,38,39] helping to link the ribosome clusters isolated in the pellet of sucrose gradients and the neuronal RNA granules observed in processes. However, many of the RBPs present in neuronal RNA granules are also found in other mRNPs and RNA granules (Figure 1). No one RBP defines the neuronal RNA granule, and the major distinction remains that only neuronal RNA granules contain 80S ribosomes [23,24].

Cytoskeletal proteins are also consistently found in the proteomics of the sedimented ribosome clusters, although some of this is due to co-sedimentation of polymerized cytoskeleton with the dense ribosome clusters, as other gradients or purification studies show a smaller amount of cytoskeletal proteins [36,38]. Nevertheless, electron microscopy findings using negative staining show that these ribosomal clusters can be associated with microtubules and actin filaments [28].

The abundance of RBPs with unstructured domains is consistent with the presumed liquid–liquid phase nature of neuronal RNA granules [29]. However, it is not clear which RBPs merely associate with mRNAs and which may play a functional role in stalling or forming the neuronal RNA granule. For instance, it was demonstrated that the transport of CaMKII mRNA to dendrites in neuronal RNA granules required the proteins Pur-α, Staufen, and hnRNP-U [38]. Still, this study did not clarify whether these RBPs were explicitly needed for the transport of CaMKII mRNA or for the transport of other mRNAs and the formation/transport of granules in general. The amount of neuronal RNA granules containing stalled ribosomes decreased after siRNA knockdown of either UPF1 or Staufen2 [39]. However, the mechanisms linking the removal of UPF1 or Staufen2 with the decrease in neuronal RNA granules remain unclear.

## What is the role of the mRNA sequences in the stalling mechanism?

**Figure 3:**
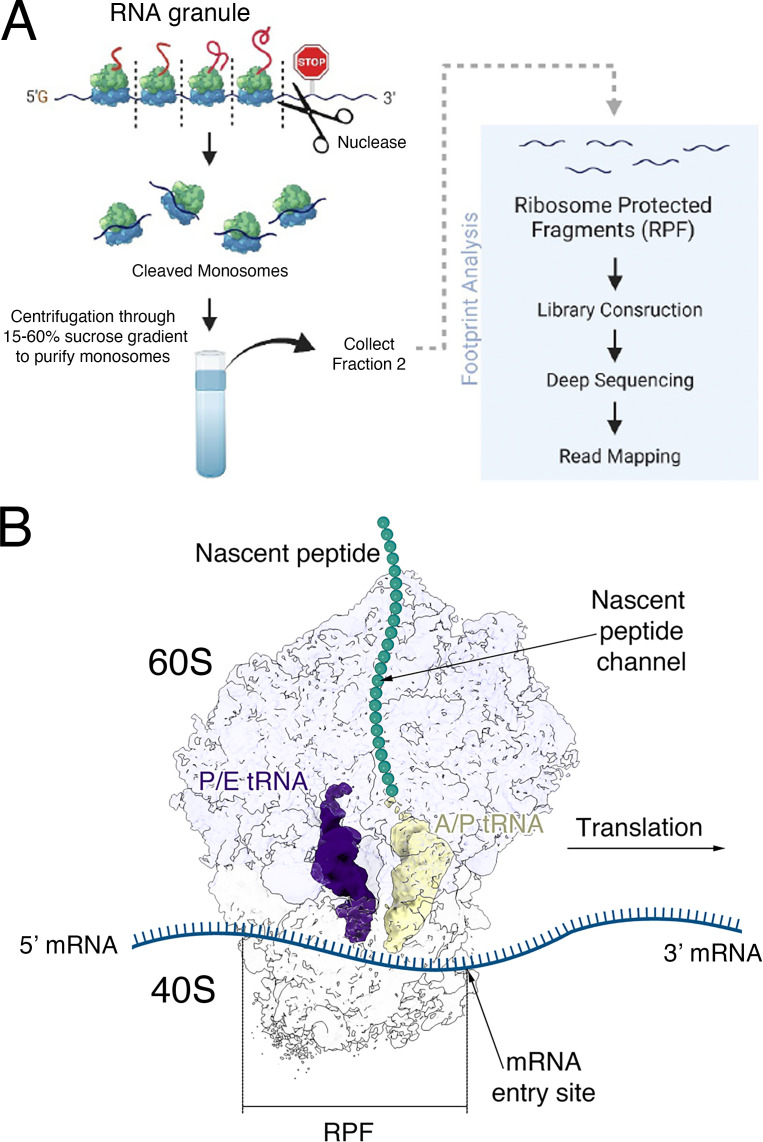
Sequencing ribosomal protected fragments. (**A**) Experimental workflow to generate and sequence ribosome-protected fragments (RPFs) from purified neuronal RNA granules. (**B**) Transparent surface representation of a cryo-EM map showing the stalled 80S ribosome from RNA granules. The image shows the location of the RPF with respect to the stalled ribosome. The image was prepared from the cryo-EM density map EMDB-29538 [27] using the ChimeraX program [40,41].

It is important to define which mRNAs are stored in neuronal mRNA granules and what aspect of the mRNA determines its inclusion. To answer this question, sedimented ribosomes from 5-day-old rat brains and ribosome profiling were used to clarify the positioning of the translationally stalled ribosomes on the mRNA molecules [27]. Ribosome clusters included in the neuronal RNA granules were isolated using sedimentation by ultracentrifugation (Figure 2) and subsequently treated with nuclease to separate them into individual monosomes. These monosomes were purified using an additional round of sucrose gradient centrifugation, and their ribosome-protected fragments (RPFs) were sequenced (Figure 3A). This study revealed that: (1) ribosomes predominantly stall in the first half of the open reading frame; (2) the RPFs show strong peaks over specific sequences in the coding region, suggesting that stalling is not random but is favored by specific sequences; and (3) these peaks were enriched in sequences previously identified in studies where fragile X mental retardation protein (FMRP) was cross-linked to mRNAs *in vivo* [42–45]. This finding aligns with a strong enrichment of FMRP in the sedimented ribosomal clusters [35,36,38], the association of FMRP with stalled ribosomes [42–46], and the localization of FMRP to neuronal RNA granules in processes [26,36]. Since the loss of the FMRP protein leads to the neurodevelopmental disorder fragile X syndrome [44,47], this implies that neuronal RNA granules are involved in neurodevelopmental processes.

While the addition of purified FMRP to ribosomes in *Drosophila melanogaster* led to a structural model of how FMRP may stall ribosomes [48], the cryo-electron microscopy (cryo-EM) map did not contain enough resolution to describe the mechanism. Also, the structure has not been seen in cryo-EM studies of the ribosomes from sedimented ribosome clusters [27]. Moreover, if the sequences in RPFs are protected by the ribosome (Figure 3B), it is not clear how FMRP can gain access to these sequences. The enrichment of these sequences in RPFs is seen even in the absence of FMRP [49], suggesting a model where FMRP associates with stalled ribosomes but does not determine where ribosomes stall. The large number of RPF sequences presents the possibility of using machine-learning analysis tools to examine whether the mRNA sequences at which ribosomes stall possess additional unique features that may contribute to the stalling mechanism [27]. However, this is yet to be explored.

### Ribosomes in RNA granules are stalled in the elongation phase of protein synthesis

Evidence using specific inhibitors has also suggested that ribosomes contained in the neuronal RNA granules are arrested during elongation. One study used either homoharringtonine, which blocks the first step of elongation, allowing ribosomes that have already passed this step of elongation to finish translation [50], or pateamine A that blocks initiation during ribosomal scanning [51]. In these experiments, extended treatment with homoharringtonine or pateamine A caused run-off of translating polysomes and prevented the formation of new polysomes. However, the ribosomes in neuronal RNA granules did not run off, indicating that they contain stalled ribosomes [26].

**Figure 4:**
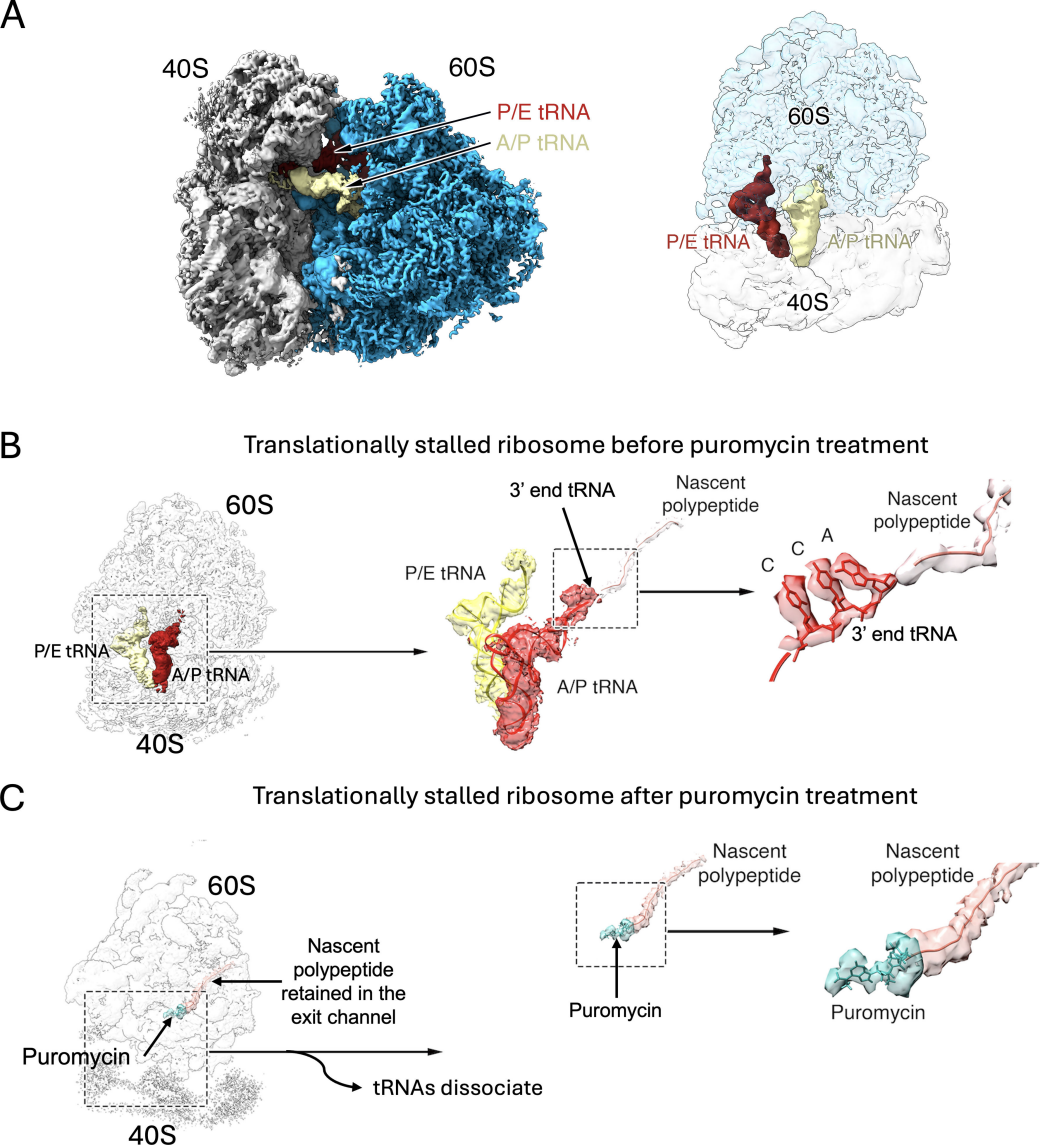
Puromycin reveals the existence of specialized ribosomes in neurons. (**A**) Side view of the cryo-EM map of the stalled 80S ribosome contained in the RNA granules (left). The right panel shows a top view of the 40S and 60S as transparent densities for viewing the position of the tRNA molecules. The image was prepared from the cryo-EM density map EMDB-29538 [27] using the ChimeraX program [40,41]. (**B**) The cryo-EM map of the stalled ribosome is shown as a transparent density to visualize the A/P and P/E tRNA molecules. The central and right panels show the tRNAs and nascent polypeptide segmented out from the map and the 3′ end of the tRNA attached to the nascent peptide, respectively. (**C**) Cryo-EM map of the stalled ribosome after treatment with puromycin. The tRNAs dissociate, but puromycin and the nascent chain remain inside the ribosome. The insets show the density for puromycin and the nascent peptide segmented out, and a close-up view of the puromycin fit into the density. Images in (**B**) and (**C**) panels were prepared from cryo-EM density maps EMDB-29538 [27] (**A**) and EMDB-40668 [55] (**B**) using the ChimeraX program [40,41].

Advances in cryo-EM and single particle analysis (SPA) approaches over the last decade [52–54] have enabled the study of stalled ribosomes within RNA granules at a molecular resolution. Two separate studies used single-particle cryo-EM analysis of monosomes isolated from ribosome clusters by RNase treatment [27,28]. This analysis confirmed that these ribosomes are stalled during the elongation phase. Most of the ribosomal particles displayed tRNA molecules in a ‘hybrid’ A/P and P/E state, which ribosomes adopt right after peptide joining (Figure 4A). The A, P, and E sites are three functional sites in the ribosome through which the tRNA molecule moves during translation. An A/P position indicates that the side of the tRNA contacting the 40S is situated in the A site, while the other end contacting the 60S is located in the P site. The same reasoning applies to the P/E tRNA state. Importantly, these experiments ruled out the possibility that elongation stalling was caused by a lack of charged tRNAs (aminoacyl-tRNAs) during purification, as this would have resulted in stalled ribosomes with an empty A site. These cryo-EM maps also allowed observation of the nascent polypeptide stalled in the ribosome exit channel (Figure 4B).

Despite the evidence that ribosomes in RNA granules are stalled in the elongation stage, the stalling mechanism remains unclear. Notably, none of the cryo-EM maps obtained in the structural studies [27,28] revealed additional densities that could be assigned to FMRP or other RBPs potentially acting as stalling factors at the tRNA-binding sites or other regions of the ribosome. The structural determination of macromolecular assemblies via cryo-EM relies on averaging thousands of particle images that represent the same structure. In this case, the presence of a low percentage of ribosomal particles with either FMRP or a specific RBP bound to them in the dataset likely leads to these densities being averaged out during the generation of the three-dimensional (3D) structure.

### Stalled ribosomes from neuronal RNA granules provide proteins for some forms of synaptic plasticity

The number of neuronal RNA granules containing stalled ribosomes is decreased by the metabotropic glutamate receptor (mGluR) agonist, DHPG. The plasticity induced by DHPG, mGluR-long-term depression (mGluR-LTD), is blocked by elongation inhibitors but not initiation inhibitors [26,39]. A form of plasticity in *Aplysia* sensory-motor neuron synapses is also blocked by elongation inhibitors, but not initiation inhibitors [56]. Thus, the proteins required for these forms of plasticity are completely provided by the reactivation of stalled ribosomes stalled in elongation stages. DHPG induces dephosphorylation of FMRP [57] and UPF1 [39], two proteins associated with the formation of neuronal RNA granules. Moreover, dephosphorylated FMRP no longer associates with stalled ribosomes [46], and preventing dephosphorylation blocks mGluR-LTD [57]. FMRP is also proposed to be important in the liquid–liquid phase structure of neuronal RNA granules, and dephosphorylation may lead to granule disassociation [58,59]. Knockdown of two other RBPs associated with neuronal RNA granules, Stau2 and UPF1, both decreases the number of neuronal RNA granules and blocks mGluR-LTD [39], further linking neuronal RNA granules to this form of plasticity.

### Are neuronal RNA granules formed with specialized neuronal ribosomes?

Puromycin is a naturally occurring compound that mimics the 3′ end of charged tRNAs. The puromycylation reaction is a standard assay to label nascent polypeptides while they are being synthesized by the ribosome. Upon puromycin treatment, the nascent polypeptide attached to the P-site or A/P-site in hybrid-state ribosomes (Figure 4B) is transferred to the amino group in the puromycin molecule [60,61]. Since the puromycylated nascent polypeptide chain is no longer attached to a tRNA, it diffuses away, translation stops, and the 60S and 40S ribosomal subunits dissociate [62]. Intriguingly, cryo-EM maps obtained from stalled ribosomes purified from RNA granules and then treated with puromycin revealed that the nascent chain is retained inside the peptide exit channel with a density corresponding to the puromycin molecule attached to its C-terminal end (Figure 4C) [55]. This has not been observed for non-neuronal ribosomes and suggests that the population of ribosomes contained in the RNA granules is structurally and functionally tailored to the unique requirements of neurons. The altered structure of neuronal stalled ribosomes is also supported by the finding that anisomycin, which competes with puromycylation in normal ribosomes, competes poorly with puromycin in neuronal ribosomes [55]. However, what makes these specialized ribosomes different from regular ribosomes remains to be determined and will require additional structural studies.

It should be noted that these experiments use a higher concentration of puromycin (100 μM) and anisomycin (100 μM) than many other studies (2–10 μM puromycin; 40 μM anisomycin)[63,64] as puromycylation of the stalled hybrid-state ribosome requires higher concentrations of puromycin than non-hybrid-state ribosomes[65,66] , and puromycylation of RNA granules is not observed at lower concentrations of puromycin [55]. Because of this, many experiments using puromycin to probe for protein synthesis do not effectively puromycylate nascent peptides in stalled ribosomes.

#### How do neuronal stalled ribosomes evade ribosome recycling?

Translational elongation can stall in all cells, and because this is a threat to proteostasis, cells have developed an extensive array of mechanisms to recognize and resolve/dispose of stalled ribosomes, collectively described as the ribosome quality control (RQC) [67] and NO-GO decay [68] pathways. When stalls cannot be resolved, ribosome recovery proteins and RQC proteins that are devoted to dissociating and degrading the nascent chains are recruited [69–71]. The NO-GO decay pathway is responsible for the destruction of the mRNA [68]. Neurons are highly sensitive to proteotoxic stress and thus are highly dependent on these pathways; mutations in RQC and NO-GO decay components contribute to neurodevelopmental and neurodegenerative diseases [72–77]. Recruitment of the RQC and NO-GO decay pathways depends on recognition of the stalled ribosome, and if neurons also use stalling as a physiological method for RNA transport, they must somehow avoid this recognition and the recruitment of the RQC and NO-GO decay proteins. Indeed, proteomic analysis of the stalled ribosomes in RNA granules [35,36] did not find any of the downstream-specific factors recruited during these responses.

One major pathway for recognition of stalled ribosomes by the cell is the collision that occurs when a following ribosome encounters a stalled ribosome [78]. The 40S–40S interaction interface formed by the two ribosomes represents a defined platform that is recognized by EDF1 [79] and the E3 ubiquitin ligase ZNF598 [71]. The subsequent ubiquitination of ribosomal protein uS10 recruits the RQC pathway [68]. However, collisions are not the only means of attracting the RQC. Stalled ribosomes in other contexts utilize different mechanisms: codon-optimality-mediated decay is recognized through the Ccr4-Not complex that detects the empty E-site [80,81]; GCN1 recognizes empty A sites and recruits a unique E3 ligase, RING finger protein (RNF) 10, along with eS10 (S3) ubiquitination [82–84]; and an occluded A site or lesions involving RNA cross-linking attract distinct ubiquitin ligases, RNF14 and RNF25 [68,85–87]

One possibility is that the compact conformation of clustered ribosomes in neuronal RNA granules renders the stalled ribosomes inaccessible to RQC and NO-GO decay factors. However, the question then becomes how the stalled ribosomes are recognized, compacted, and assembled into neuronal RNA granules. Since cells have many ways of distinguishing ribosomes based on their state, but none are specific to the hybrid state, neurons may have developed a unique way of identifying stalled ribosomes based partly on recognizing hybrid-state ribosomes. The P/E site, in particular, may be an attractive recognition site, and cross-linking studies of G3BP2 show binding near this region in stalled ribosomes [28]. Moreover, UPF1, which normally associates with ribosomes at the stop codon through binding ERF1, also binds near the E-site in ribosomes independently of ERF1 [88], and loss of UPF1 decreases the number of RNA granules containing stalled ribosomes [39]. Identifying how neurons recognize stalled ribosomes and package them into neuronal RNA granules is a critical question for future studies.

Cryo-electron tomography is ideally suited to determine how stalled ribosomes are organized within ribosome clusters in neuronal RNA granules [89]. Solving the structure of individual ribosomes using SPA cryo-EM approaches requires collecting thousands of images of these ribosomes from different view angles and computationally averaging similar views to combine them into a 3D cryo-EM structure. Since averaging is an essential part of the structural determination, the premise for SPA cryo-EM is that images of the ribosomal particles contributing to the cryo-EM map represent the same structure or functional state of these ribosomes. This premise is not met when attempting to obtain the 3D structure of an entire ribosome cluster. Ribosome clusters in different RNA granules contain a different number of ribosomes, and each ribosome likely relates to other neighboring ribosomes in the cluster in a variable manner, making SPA cryo-EM approaches inadequate. Instead, in cryo-electron tomography, the vitrified grids containing the RNA granules are used to collect a single-tilt series of electron microscopy images between −70° and +70° that are combined through back-projection to produce a ‘tomogram’ or 3D structure of the entire RNA granule. No averaging is applied in this process. The analysis of these tomograms can determine how the stalled ribosomes associate with each other and what supramolecular arrangements are formed in the context of the clusters and the neuronal RNA granules. This information will also be essential to understanding the architecture of the neuronal RNA granules and how their higher-order organization prevents the triggering of the protective RQC and NO-GO decay responses seen in other cells.

### Remaining questions and future outlook

A critical question is whether neuronal RNA granules are a dense collection of monosomes or polysomes, or if they have a different structure altogether. It has recently been proposed that most local synthesis in neurons is mediated by monosomes [90]. Indeed, monosome stalling may reduce the possibility of collision-mediated recognition of stalled ribosomes. mRNAs identified as predominantly translated by monosomes in neurons are enriched in the sedimented clusters of ribosomes from RNA granules [27]. These ribosome clusters required more nuclease than is typically required for polysomes to be converted into monosomes [27,28,44]. Moreover, the sedimentation of the ribosome clusters from neuronal RNA granules is sensitive to salt and detergent in ways that polysomes are not [36]. Therefore, it is likely that the ribosomal clusters in RNA granules are not *bona fide* polysomes, but rather, monosomes associated into dense clusters through a distinct mechanism. Additionally, canonical polysomes of actively translating ribosomes contain only a single mRNA molecule threaded across all the ribosomal particles in the polysome. In the case of RNA granules, it remains unknown how many mRNA molecules are included in the ribosome clusters. Cryo-electron tomography and other advanced cryo-EM techniques will be critical in providing insights into the number of mRNA molecules contained within an RNA granule. In general, understanding what holds stalled ribosome clusters together is one of the most significant outstanding questions relevant to how stalled ribosomes can act as a transporting structure in neurons.

How is reactivation of mRNA translation and protein synthesis mediated? Is there a mechanism to efficiently restart translation on an mRNA still present in the neuronal RNA granule, or is dissociation of the granule required before reactivation of translation? Will stalling occur locally at synapses after reinitiation of these mRNAs? If stalling occurs locally, will this result in repackaging into granules?

In the early brain and hippocampal cultures, most nascent chains are resistant to run-off [26], and most ribosomes show anisomycin-resistant puromycylation [55], suggesting that the majority of the nascent chains are on stalled ribosomes. Due to the challenges in isolating ribosomes by sedimentation from adult brains due to myelination [37], it remains unclear how levels of RNA granules change throughout development. Finally, other than mGluR-LTD [26,39], it is not clear what role the proteins produced after reactivation of stalled ribosomes play in neurodevelopment, plasticity, and homeostasis. Until the molecular underpinning of stalling is better understood, it will be difficult to specifically block only translation coming from reactivation of stalled ribosomes and thus determine all of the contributions of stalled ribosomes to the physiological roles of local protein synthesis.

**Perspectives**

- Local translation in neurons is a key neuronal-specific process critical for neurodevelopment and synaptic proteostasis; however, the molecular mechanisms remain poorly understood.
- A critical feature of local translation is that specialized neuronal ribosomes are stalled in elongation, organized into ribosome clusters, and transported to synapses in a neuron-specific RNA granule.
- Structural and biochemical studies are needed to determine how the neuronal ribosomes are specialized for stalling and how they escape normal ribosome quality control.

## Abbreviations

FMRP: fragile X mental retardation protein
P bodies: processing bodies
RBPs: RNA-binding proteins
RNF: RING finger protein
RPFs: ribosome-protected fragments
RQC: ribosome quality control
SPA: single particle analysis
cryo-EM: cryo-electron microscopy
mGluR-LTD: mGluR-long-term depression
mRNPs: mRNA-containing ribonucleoproteins
